# “Binge eating disorder is the slum of eating disorders”: a qualitative study of Norwegian women with binge eating disorder in the encounter with the healthcare system

**DOI:** 10.1186/s40337-025-01223-z

**Published:** 2025-03-19

**Authors:** Julie Riise, Kjersti Solhaug Gulliksen, KariAnne Vrabel, Margrethe Seeger Halvorsen

**Affiliations:** 1 The Institute for Eating Disorders, Oslo, Norway; 2 The Norwegian Directorate of Health, Oslo, Norway; 3 Research Institute of Modum Bad, Vikersund, Norway; 4 Department of Psychology, University of Oslo, Oslo, Norway

**Keywords:** Binge eating disorders, Treatment needs, Qualitative study, Patient perspectives, Healthcare experiences

## Abstract

**Background:**

Binge Eating Disorder (BED) is the most prevalent eating disorder, yet it remains under-recognized and insufficiently understood in both healthcare and society. This leads to a lack of appropriate treatment options and challenges of identification within somatic healthcare. Our study aims to elucidate effective treatment approaches for BED by exploring patients’ personal understandings of their treatment needs.

**Methods:**

We interviewed 6 individuals diagnosed with BED regarding their healthcare experiences and analyzed the data using a modified qualitative method combining thematic and interpretive phenomenological analysis.

**Results:**

The analysis resulted in three main themes: Lack of understanding, Trapped in body shame and Hope and movement, each with belonging subcategories. These themes narrate a journey from being unrecognized with a psychological issue, feeling immobilized by body shame towards embarking on recovery. Particularly Trapped in body shame links the other main themes representing a barrier and a pivotal point in the recovery process.

**Conclusions:**

Our study highlights that shame related to binge eating and body image is pervasive in participants, exacerbated by a healthcare system that often prioritizes weight and lifestyle. Such shame can block treatment access and prolong the disorder. We argue for a paradigm shift in clinical practice towards patient-centered care that prioritizes empathy and holistic support over weight-focused models. Group therapy can be beneficial in reducing shame, if the group composition is carefully considered. Effective BED treatment should involve creating a safe environment for discussing body shame, emphasizing the need to address this issue to improve treatment effectiveness and patient satisfaction.

**Supplementary Information:**

The online version contains supplementary material available at 10.1186/s40337-025-01223-z.

## Introduction

Binge eating disorder (BED) is the most common eating disorder (ED) with prevalence rates ranging from 0.2 to 6.6% of the worldwide population [1–3], yet its severity seems insufficiently recognized [4]. The number of people presenting for evaluation, receiving a BED diagnosis and requiring treatment are expected to increase [5, 6]. BED is frequently associated with psychological and somatic health problems, impairment in functionality regarding school/work, family and social life, and health related quality of life [7], leading BED to be a costly diagnosis representing remarkable health impacts and economic costs to society as well as individual suffering. Despite its prevalence and severity, awareness and knowledge about BED is inadequate in healthcare and in the general population. Treatment offers are scarce, and BED is likely under-identified in somatic healthcare. This study seeks to increase the understanding of what might be important considerations in BED treatment, by investigating patients subjective understanding of their own needs in treatment.

Limited knowledge exists on which interventions for BED may be more effective than others in treatment. Meta-analyses comparing treatment effects, include different aspects of clinical outcomes, including binge eating behaviour and related psychopathology, weight loss and appetite [8, 9]. A meta-analysis including 28 treatment comparisons found some significant differences between pharmacological interventions, psychotherapy and behavioral weight loss on different aspects of outcome, while the majority of treatment comparisons revealed few significant differences between groups [8]. Eating disorder-focused Cognitive Behavioural Therapy (CBT-ED) is commonly recommended as a first-line psychotherapy treatment for BED by National Institute for Health and Care Excellence [10]. However, research show that up to 50% of patients with EDs, including BED, do not respond sufficiently to recommended treatment interventions like cognitive restructuring [11, 12] and do not obtain remission with psychotherapy treatment alone or in combination with weight loss medication [13]. This is especially true for patients exhibiting more self-criticism and shame and has led to the development of treatments that specifically address self-compassion as a way to support these individuals [12]. Furthermore, recovery rates largely depend on how recovery is defined [14]. There is a need for better understanding of mechanisms that promote change, in light of the generally low response rates for treatment.

Surprisingly, less than half of patients with BED receive any form of treatment [2] and there is limited understanding of treatment satisfaction and how to increase treatment participation [15, 16]. This is important to understand more extensively, as we know from research and clinical practice that faltering motivation and high drop-out rates are common in ED treatment across diagnostic categories [17–19]. The purpose of this study is therefore to enhance our understanding of how patients with BED experience their encounters with the healthcare system and how they experience their treatment needs by incorporating patients’ subjective perspectives.

Several factors may explain the gap between the high BED prevalence and the low number of patients receiving BED specific treatment. In the Norwegian context of this study, psychotherapeutic treatment offers are close to non-existent, as BED is not included in ICD-10, the official diagnostic manual guiding patients’ rights to treatment in public healthcare. Furthermore, BED is associated with psychiatric and somatic comorbidity such mood disorders, anxiety, substance abuse [20], and obesity, diabetes type 2, hypertension, chronic pain and respiratory diseases [1, 21–24]. This may lead treatment to be aimed at comorbid conditions rather than on BED itself. People with BED are 3–6 times more likely to have obesity than people without an ED [2, 20]. Among people who seek weight control treatment, the prevalence of BED is estimated to be 30% [25]. Additionally, BED is more occurrent among people awaiting bariatric surgery than in the general population, with estimates varying considerably between 4.2 and 47% [26, 27]. Post-operative grazing, which can mask underlying BED, has also been observed among patients who have undergone bariatric surgery [28]. These factors indicate that cases of BED may often be overlooked within somatic healthcare and weight management settings, with little attention given to the psychological aspects of BED.

There is lacking knowledge of the clinically significant psychological characteristics of BED [29–31], that may inform the development of treatment interventions. Research suggest that feeling inferiority due to weight stigma and adverse childhood experiences seem to keep patients with BED in a shame driven pattern of dieting, weight loss, bingeing and weight regain [32]. Weight stigma refers in this context to experiencing the body as target for weight bias in healthcare settings, in professional and personal life, and experiences with critical comments and dieting encouragement from significant others from an early age. Adverse childhood experiences refer to traumatic events such as violence, sexual abuse, bullying and fear of alcoholic parents or partners, that BED patients see as having influenced a negative relationship with their bodies [32]. Unlike in anorexia nervosa (AN) and bulimia nervosa (BN), body image disturbance is not considered a diagnostic marker for BED. However, body overvaluation and dissatisfaction are associated with BED, and the degree of body dissatisfaction and overevaluation indicates greater severity and course of the disorder [33–39]. Furthermore, binge eating is often accompanied with experiences of shame [40, 41]. Given the association between BED and overweight, it is relevant to take into account the documented associations between weight bias, morbidity and mortality [42–44]. Self-reported experiences of shame often serve as a deterrent for individuals with EDs, including BED, to seek treatment [45]. The way in which these patients experience shame may contribute to the generally low efficacy of treatment and could inform the improvement of patient care in clinical settings. We know less about how shame manifests in BED patients compared to other EDs, however research suggest that body shame and weight stigma seems important to understand in association with BED specifically [32]. Therefore, the subjective perspective of BED patients is relevant for expanding knowledge on this topic.

Studies have shown that individuals with EDs experience complex perceptions, thoughts and emotions in their initial interactions with healthcare professionals, which may potentially impact the therapeutic relationship and process [46]. For patients with BED, feelings of shame and self-criticism related to eating behaviour and body are particularly prominent and may contribute to delayed help seeking, reluctance to engage in treatment and relational obstacles in initial encounters with healthcare professionals. For BED patients specifically, these challenges are often compounded and amplified by a lack of awareness and understanding of BED among general practitioners and mental health professionals, who may attribute symptoms to comorbid conditions. Historically BED has been overlooked compared to other EDs and limited access to specified treatment options have further left patients without recognition and adequate care, potentially reinforcing barriers to safe treatment alliances within the patient in a treatment setting. Furthermore, research suggest that experienced health professionals within the field of EDs report less enjoyment and confidence in treating BED compared to AN and BN [47], underscoring potential challenging links between BED patients´ experience in the therapeutic relationship and practitioners’ approach in early encounters.

From the patients’ perspective, supportive and understanding relationships with health professionals and persons outside treatment are considered important for recovery [48–50] and for the help-seeking process [51]. For BED patients particularly, this may mitigate experiences of judgement and stigma. Supportive relationships extend beyond healthcare providers and may include family and social network that is often strained by the psychosocial consequences of BED, such as social secrecy and withdrawal. These factors can play a crucial role in recovery and help seeking behavior for these patients, who may prioritize interventions that address relational and emotional aspects of their disorder. Preliminary work in the field of EDs suggests that treatment satisfaction is closely associated with the way treatment is delivered, regardless of the treatment modality [48, 52]. To our knowledge, this is not investigated for BED specifically and should be explored further.

### Aims

In the summary above, key issues are highlighted regarding the treatment gap for BED, unsatisfactory treatment outcomes and the need for a better understanding of this clinical phenomenon to improve and tailor treatment offers. Furthermore, there is a lack of qualitative studies examining the subjective experiences of patients with BED and their perspectives on the care they receive. To address this gap, this study explores nuances of patient experiences in relation to help seeking and treatment satisfaction, using in-depth qualitative analyses.

To our knowledge, existing qualitative studies of BED focus on quite specific aspects such as gender differences [53], addiction [54] and general experiences of having BED [55]. A few qualitative studies investigate specific treatment approaches [11, 32, 56, 57] and recovery facilitators [50]. To our knowledge there are no qualitative studies that specifically investigate general healthcare experiences and subjective needs in treatment for BED patients. This study does not compare methods of intervention or specific treatment approaches. This study explores how individuals with BED perceive their needs in treatment across treatment interventions and health care approaches and examines how their experiences can inform us of how these needs are met within both the general and specialist healthcare system. An exploratory qualitative method is therefore considered appropriate.

The study will investigate:

- How do individuals with BED perceive their needs in treatment?
- How do their experiences in healthcare relate to their specific needs in seeking and receiving treatment?

By exploring these questions, the study aims to provide novel insights into the patient perspective on treatment for BED and inform improvements in the quality of care provided. The study will adopt a qualitative method to capture detailed subjective descriptions of patient experiences, with the aim of enhancing the care BED patients receive based on their perspectives.

## Method

### Sample

The study included 6 Caucasian females, between age 28–50, who were recruited through two facilities giving either specialized inpatient treatment for BED or a psychoeducative group intervention for BED. These facilities were among the very few places that provided any form of BED specific help in Norway and they were both pilot programs, as the first of its kind at each facility. There were no inclusion criteria regarding what specific kind of treatment or help the participants had undergone, given a general lack of treatment offers for BED prior to this. The inclusion criteria encompassed individuals of 18 years or older, diagnosed with current or prior BED and assessed by their clinician to ensure the capacity to provide informed consent.

The first author (JR) contacted clinicians of the facilities who asked their patients for consent to be contacted and further informed of the study by JR. Participants who concurred to this were then recruited by their clinician and asked for consent to participate in the study. Four participants were recruited from a specialized inpatient treatment for BED including meal support, group based physical activity and individual psychotherapy over 6 weeks. Two participants were recruited from a specialized ED treatment facility giving psychoeducational group intervention for BED over 10 weeks, held by a clinical psychologist and a clinical dietitian. The treatments at the two sites are not directly compared in this study and served only as a means to recruit patients with a BED diagnosis who had experiences within the healthcare system.

The participants were asked to answer a brief questionnaire about their mental health status and experiences with psychiatric treatment in general before the interview. None of the participants had previously received a formal BED diagnosis, while all of them reported struggling with an undefined eating disorder for many years prior. The actual duration of their BED in clinical terms is therefore unknown. All participants either was or had recently undergone other forms of psychiatric treatment in addition to the treatment they received for BED, at the time the interviews were conducted. All participants had other psychiatric conditions in addition to BED, including anxiety, depression, PTSD and personality disorders. All participants had experiences with seeking help for binge eating in both primary healthcare and specialized care, although this was not an inclusion criterion. All participants had finished higher education. Two were working full time, two worked part time and two received public welfare (see Table 1).

**Table 1:** Participant characteristics, N = 6

Participant characteristics		Number
Gender	Female	6
Ethnicity	Caucasian	6
*Age*		
	20–30 yrs	1
	31–40 yrs	4
	41–50 yrs	1
Educational level	Higher education (minimum BA)	6
Employment status	Full time employed	2
	Part time employed	2
	Public welfare (100%)	2
Self-reported onset of eating disorder	Pre-teen	6
Psychiatric history	Previous treatment in specialized psychiatric care (for any disorder)	6
	Ongoing psychiatric treatment	3
*Other diagnoses in addition to BED*		
	Anxiety disorder	3
	Depression	5
	Personality disorder (any)	2
	PTSD	3
	OCD	1

### Reflexivity

The research team is comprised of four female, Caucasian, Norwegian speaking psychologists. All are clinical psychologists with considerable expertise in treating a variety of EDs across multiple treatment environments and represent different theoretical orientations, i.e. psychodynamic, cognitive behavioral, compassion focused and integrative perspectives. Among the team members, three possess academic qualifications: Two as associate professors, and the third holds a PhD in clinical psychology. Both the homogeneity and the diversity in our personal, professional and academic backgrounds fostered a reflective approach that was proactively integrated throughout the research process. We engaged in self-reflection of our experiences and theoretical perspectives and tried to be aware of how our assumptions may influence the analytic process. To enhance the trustworthiness of findings, the researchers met regularly for consensus meetings to discuss interpretation of the data material and develop the thematic hierarchy (fidelity/ methodological integrity [58]_._

### Setting and procedure

The interviews were semi-structured and conducted by the first author (JR) using an interview guide developed in cooperation with the last author (MSH) (see supplementary material). The interview guide included questions on three main topics; the participants’ experiences of having BED and other possible mental health issues; experiences with healthcare in relation to their BED, and lastly; the participants perspectives on, and potential experiences of, individual recovery in relation to seeking help. Lastly, a segment assessing the participants perceptions of salient aspects to emphasize was included. The interviews were conducted in-vivo in suitable locations at the Department of Psychology, University of Oslo. They were audio-recorded and transcribed verbatim by the first author.

### Analysis

We utilized a modified approach combining reflexive thematic analysis (TA) [59, 60] and interpretive phenomenological analysis (IPA) [61] to analyze our data. The analysis was guided by both approaches throughout the analytic steps described. Initially, the first author (JR) engaged deeply with the data through listening to recordings, verifying transcriptions and multiple readings, annotating insights relevant to the research question. Initial descriptive, linguistical and conceptual notes were taken according to IPA [60]. From this, themes were inductively created employing a bottom-up approach. All authors collaboratively reviewed and refined emergent themes, while the third author (KAV) acted as an independent auditor, ensuring robustness in the analysis. Chronologically organized coding within interviews preserved idiosyncratic details while also allowing for the abstraction of patterns across the dataset.

In the following phase, JR grouped codes into categories to isolate potential main themes, engaging in continuous discussion with co-authors MSH and KSG, and later KAV, for a comprehensive evaluation. We strived to capture overarching patterns representative of the entire material, iterating between abstraction and subsumption. To affirm the integrity of thematic labels, text abstracts were consistently re-examined following hermeneutic circularity principles. The potential main themes underwent systematic organization and a review process, involving refinement, exclusion, or renaming as appropriate. This meticulous approach facilitated identification of a set of main themes for each interview, ensuring an ideographic focus whilst distilling commonalities across cases.

The final stage involved analyzing the collate lists of potential main themes to discern cross-case patterns. We transitioned from the case-specific structure to a broader outlook, irrespective of hierarchical theme classification within cases. The primary researcher JR initially led the analysis regularly consulting co-authors for insights. Our thematic exploration was kept wide-ranging to accommodate the nuances of participants experiences with BED, while still prioritizing themes rich in descriptive content, and those demonstrating similarities or differences. This led to the identification of three comprehensive main themes with belonging subcategories, which were interpreted as interconnected dimensions rather than isolated constructs.

## Results

Three main themes were developed from the material.

**Fig. 1:**
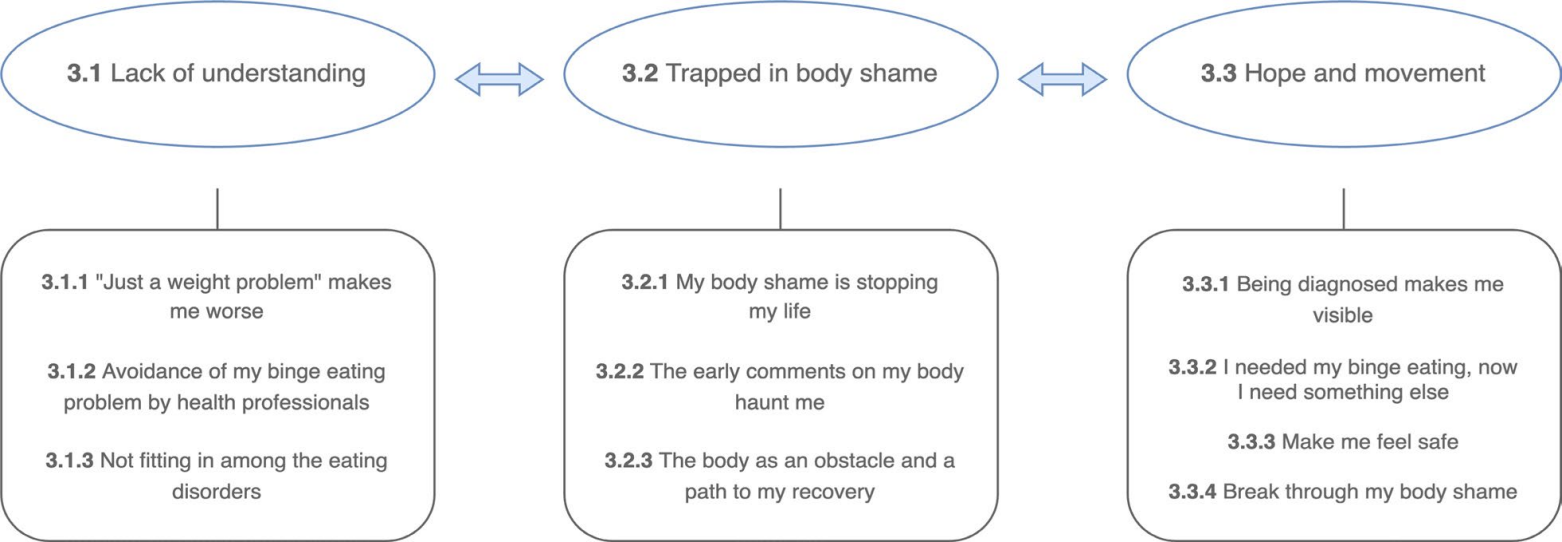
Main themes and subcategories

1. Lack of understanding
2. Trapped in body shame
3. Hope and movement

each with belonging subcategories (Fig. 1). Categories are further illustrated below by quotes from individual participants. Quotes have been assigned pseudonyms to secure participant anonymity. The main themes represent a movement through conditions of having a psychological health issue that is not understood, feeling stuck in the body, towards positive movement or start of a recovery process. The main theme **Trapped in body shame** binds the two other main themes together, as it represents both the experience of standing still in their condition and a specific way through which a recovery process can take place. Body shame has to be overcome to allow positive directed movement (see Fig. 1 for illustration).

### Lack of understanding

A main theme as it seemed to reflect all participants experiences with meeting healthcare in significant ways. The subcategories illustrate some ways participants felt misunderstood: as just having a weight problem (Sect. Just a weight problem makes me worse), by their binge eating being avoided by practitioners (Sect. Avoidance of my binge eating problem by health professionals), and by not feeling like they fit in among the EDs in treatment (Sect. Not fitting in among the eating disorders). Feelings of shame was induced or reinforced in all ways of not being understood by healthcare practitioners.

#### "Just a weight problem" makes me worse

A subcategory expressing one way of being misunderstood. All participants shared experiences of being understood as primarily having a weight problem. Everyone had several years of degrading experiences of seeking help for their bingeing and receiving weight-reduction focused interventions that triggered the binge eating and worsened their condition. A general experience of practitioners seeing their eating problem as something reflecting personal character had for some participants become an internalized attitude towards themselves. To others, it became a reductionistic contrast to their own understanding of their own problem.

Hanna: ´*Have you considered bariatric surgery?'. I don’t know how many times I have heard that. And I just… can’t have bariatric surgery because I will kill myself. And they don’t understand that, because they think that if you just have a smaller stomach your brain will change*´.

Being understood as having a weight problem and met accordingly with weight reduction interventions seemed to work against the underlying wish for recovery by triggering the binge eating and inducing shame for not being able to gain control. This created externally induced feelings of hopelessness in their help seeking efforts.

#### Avoidance of my binge eating problem by health professionals

This subcategory reflects how participants experienced that their binge eating was not addressed in the same manner as other psychological problems when seeking help in psychiatric healthcare. However, several of the participants experienced their BED as the most urgent among several issues such as depression, PTSD or anxiety.

Maya: ´*It´s fascinating how much help I have been seeking for binge eating and how much help I have received for everything else. It´s like they are looking for something else to avoid focusing on the bingeing*´.

#### Not fitting in among the eating disorders

This subcategory reflects how the participants longed to be understood as having an ED and compared themselves to restrictive forms of EDs such as AN and BN. A feeling of being on the outside and of being marginalized in treatment was present when they saw the way the healthcare system treated these forms of EDs compared to their BED. Several participants wanted a diagnosis for their binge eating but found that a part of their ED was «missing» through lack of compensatory behavior or low weight. In this way they experienced being perceived as having an incomplete form of ED. Being de-prioritized in ED treatment based on their symptom presentation added to this experience. When being included in treatment for ED, an experience of being on the outside remained. This was partly based on a bodily experience.

Emily: ´*I was very grateful for being included there because they understood that I had an ED, but it was extremely difficult to be in a group with so many skinny people. I was the only one who was big and then they talk about having to gain weight. (…) I think they have to address the fact that we are bigger and do not have the same ED as a restrictive eater, and that we feel an enormous shame. If not, there is no help´*.

Anna: ´*I felt like we were the big ones who didn’t even through up, we were the failures who shouldn’t be there*´.

Being taken less serious with their ED symptom presentation seemed to add a layer of shame of the binge eating, and a feeling of having a failed ED. This was reinforced by the lack of treatment offers in the treatment system and poor understanding of the clinical traits of their disorder.

### Trapped in body shame

The main theme Trapped in body shame reflects how all participants described feelings of body shame as blocking their life experiences and recovery. The body shame was present in relation to healthcare practitioners in a way that hindered good treatment alliances. A feeling of being «trapped» in this body shame seemed remarkably present. Trapped in body shame is therefore directly linked with Lack of understanding as a way their condition is maintained and as the existing basis for Hope and movement.

#### My body shame is stopping my life

This subcategory describes how the participants experienced that body shame was blocking their life participation and how this expanded across important aspects of life to them. The participants expressed awareness of the extent of their body shame, while at the same time it seemed to be a tormenting normal baseline for their life. The body's significance for self-evaluation was prominent and became like a precondition for living. The body shame led to withdrawal from activities and social isolation and became something that the participants felt forced to adjust to in severe ways.

Hannah: ´*I do all I can to avoid it (my body). I can’t move back to my hometown because there are so many big windows. So, I live in (name of town), because there’s just the woods here. There are so few stores and things to be exposed to. So, I have lived here for eight years, just to get away. (…) I feel like it's my body that is stopping my life. And I have a man who I like and who I’have had contact with for ten years. And he is a very good person, but I just can’t take it further with him*´.

Most of the participants described experiences of being corrected and receiving comments on their eating and body from significant others early in life. 

#### The early comments on my body haunt me

Express how participants saw the impact from this on their relationship with food, their body and with themselves.

Anna: ´*I am disgusting… So, I can just be a trashcan for everything disgusting. (…) When we had cooking in primary school, they used to say 'just send the waste to Anna, she’ll eat it'. And I was bullied a lot… And at home it was like 'just eat this, so we don’t have to through it out'. And at the same time yelling at me because I ate too much*´.

The participants were aware of how these experiences of commenting by significant others have impacted their BED development. The comments on food and body still influenced the way the participants evaluated themselves through their body and eating behavior.

#### The body as an obstacle and a path to my recovery

All participants talked about their body when asked about their experiences of recovery. This subcategory expresses how participants describe recovery from their BED as closely connected to the relationship with their body. A negative relationship with their body was experienced as an obstacle for recovery. Recovery therefor entailed a more positive or neutral body image to them. To some, body shame was in itself experienced as a sustaining factor for the binge eating. The participants also addressed losing weight in relation to recovery. To some, a conflict was experienced between wanting a more accepting relationship with their body and a wish to lose weight at the same time. Some described that a wish to lose weight was driven by the ED and recovery entailed becoming free of this wish. To others, losing weight and gaining a more positive body image was less separated as pathways to recovery.

Mariel: ´*On the one hand recovery is that I stop bingeing… and not having to think about food 24/7, that’s recovery in one way. But then there is this other part of me that’s driven by the ED and by a chase for like… The first thing that came up in my head when you asked me that question wasn’t 'maybe I get to feel better and get to be free from thinking about food', it was 'maybe I get to be skinnier? Maybe I get to be a better version of myself, and maybe I finally get that control…'. It’s like this war in my head all the time about what recovery really is*´.

I: ´*What is recovery to you*?

Anna: ´*Well, I’m not recovered. So, to me that means reaching a normal weight. And to be able to have a relationship with my body at all so that I can do the things I want to do. Like, if I want to go buy new clothes, I can do that. Or going out with someone without having to go home right away or cancel because I was doing my makeup and looked in the mirror and discovered I have a body too. I just feel like if my body looked normal, I would be able to fake it in a way… But I don’t know… like If I lost 50 kilos, I don’t know what my life would be like… But at least people wouldn’t see me the way I feel they do*´.

To Anna, losing weight was less separated from recovery. At the same time, she reflected on whether it represents a deeper or a complete way of recovery.

### Hope and movement

The main theme **Hope and movement** entails the participants experiences of movement towards recovery. This main theme is therefore a contrast to previous experiences of being stuck in shameful states in the other main themes. The subcategories entail different ways of positive and changing experiences in the participants meetings with healthcare. By being seen and met for their BED, they seemed to develop new ways of seeing themselves and their problems. When their previously unseen BED was recognized and exposed, it entailed a vulnerability especially connected to feelings of shame. The subcategories reflect ways to tolerate or reduce this shame.

#### Being diagnosed makes me visible

This subcategory expresses how getting a description of what they struggled with through a diagnosis affected all participants in significant ways. To some participants, having their problem defined by a diagnosis was linked to feeling recognized and not being as alone with their struggle. This contributed to a stronger self-recognition of the problem as well, that lead to a realization of having to create change in a different way than before.

Emily: ´*I remember crying… out of joy and out of sorrow in a way… I felt like now it was true, and someone is listening and that’s really scary cause then its real, but it felt really beautiful too. But I was really… really sad for myself. (…) It just hit me in the chest really hard. Because now it's been recognized, you know it's your job to make it go away now*´.

To be recognized through a diagnosis seemed to be experienced as a relief from guilt and shame. To others, getting the diagnosis evoked feelings of shame in relation to how others may perceive the diagnosis.

Isabel: ´*I don't think it’s cool to have that diagnosis at all. It's like the slum of eating disorders. I mean, not that it should be cool to have a diagnosis, its fine, but.. because then you have a proof that you have an ED, and if that’s what it took for me to get help, that’s good. But I see my ED as the slum of eating disorders*´.

The participants’ experiences of being diagnosed were ambivalent and associated with how the diagnoses was perceived by themselves and by others. By reducing feelings of guilt and shame and by having to deal with the problem in a new way, being diagnosed created way for positive change.

#### I needed my binge eating, now i need something else

This subcategory sxpresses how the participants gradually understood their eating problem as an expression of underlying struggles and that food had become a way of handling difficult emotions. In contrast to feelings of guilt and shame, this seemed to contribute to self-compassion. Additionally, it seems to facilitate inner work towards finding alternative ways of handling their underlying struggles. To some, recognizing this also entailed challenges in defining the underlying issue and started a process of looking for causes and mechanisms behind the symptom behavior.

Maya: ´*Everything has been there as a security. Like my self-confidence, my comfort, my strength, my secret weapon. If you hurt me, I can hurt myself even more, I can eat even more candy so that I don’t have to feel how you caused me pain*´.

Isabel: ´*When I fix the symptom and change how I eat, the anxiety just becomes stronger and stronger. And I can do that for a while, but then it just bursts (…) When I don’t understand what’s making me do this, I don’t know what problem to actually fix. So, it became clear to me that I’m like… someone who uses food to handle things I've experienced. Before I used to think that food was controlling my emotions, but now I understand that my emotions are what’s actually controlling my eating*´.

The participants described recognizing the coping strategy food represented in their life as a starting point for recovery. Food seemed to fill a space that could be replaced or changed for the participants when they understood the quality of its function.

#### Make me feel safe

This subcategory describes how participants experienced the prerequisites for safety and trust in the treatment relationship as defining for positive movement, but also as very fragile. When openness about something that earlier had been unsafe to talk about was facilitated, movement towards recovery occurred. The fragile prerequisite for trust could be seen in relation to shameful experiences and earlier encounters with the healthcare system where trust was either not present or broken.

Hannah: ´*I felt like talking to the other patients helped me more than talking to the psychologist. To me, being able to actually sit there and open up to others and relate to them… and feel like I’m not alone, I’m not stupid, I’m not… for not being able to control this. It’s not like I failed at something. And also feeling the responsibility of contributing to the group. In that way I was kind of forced to open up like the others did. Because when it helped me so much to listen to them, I had to contribute if that could help them as well*´.

Mariel: ´*When I’ve been to DPS [i.e., public psychiatric healthcare] I’ve just been able to steer the conversation so that I escape all the uncomfortable questions. No one says 'How so?' or 'Say more about that?' (…) It’s my own fault that it happens, that I don’t choose to bring it up, but it might be that I think it’s too hard to do that. So, I feel like at (name of treatment facility) was a turning point for me, because I felt like they took me very seriously. And saw more behind what I was presenting at once*´.

The need to be seen for their binge eating was significant, and simultaneously a conflict to the vulnerability felt in exposing something shameful. It seemed clear that participants needed to be helped to expose this and that safety had to be facilitated by another person.

#### Break through my body shame

This subcategory expresses how talking about the body in the treatment relationship could make way for positive movement. This subcategory can be seen as directly connected to *The body as an obstacle and a path to my recovery* and *Make me feel safe.* Feeling body shame and simultaneously experiencing that healthcare professionals did not address the participants body experience, reinforced body shame for the participants. Body shame was blocking a safe treatment relationship and recovery, but seemed to be something that was possible to overcome by talking about it. It seemed that this pathway needed to be facilitated from the outside and that the participants needed help with this.

Isabel: ´*It's the only thing I feel like we don’t talk about. Even in ED treatment or anywhere really. And what’s even more difficult is that they do it with skinny patients… (crying). So, it just signalizes that it’s really shameful*…´

Anna: ´*It's like the therapists avoids it too. But if you present something that signalizes «don’t go there», then… But I don’t think I can recover without being able to relate to it (my body). But I don’t know how. I think I need to be pushed into it, because I understand that it is the only thing I need to fix, but it's so hard to start talking about it, because I don’t know when or how or.*..´

When body shame was not addressed in treatment it seemed to confirm a feeling of having a wrong kind of body, a body that was not an approachable theme in treatment for the participants. Isabel described how this feeling was reinforced by observing how therapists did this when treating underweight patients. Anna pointed to a possible contribution of her own for not addressing it, which shows how difficult it could be to open up about this from the patient perspective. The need to address the participants body experience and to receive external help to talk about it was clear through the participants descriptions.

## Discussion

This study aimed to explore the needs and treatment experiences of individuals with BED within the healthcare system. The main findings revealed that patients experience significant internalized shame related to their binge eating behavior and body image. This shame, often exacerbated by the focus on weight and lifestyle in healthcare, acts as a barrier to seeking treatment and sustains the disorder. Participants reported that this shame was rooted in early life experiences and reinforced through interactions with healthcare professionals.

### Encountering the healthcare system with shame

The findings indicate that participants carry an internalized shame and experience shame specifically related to their binge eating behavior and body image when interacting with the healthcare system. Participants anticipate that others will judge their problem as something shameful. The results show how shame has been externally imposed and internalized early in their lives. As a comping strategy, the participants withdraw and isolate themselves to escape the shame felt in social contexts. Seeking treatment requires participants to expose themselves to situations that activate and reinforce these feelings of shame, posing a barrier they must overcome.

Shame is associated with psychopathology [62–65]. Its relevance in ED pathology is well documented [40, 41, 66–70]. The results in our study indicate that the extent and degree of shame participants experience is pathological and closely related to their BED as both an underlying mechanism and sustaining factor*.* The role of shame is therefore highly relevant when understanding the participants needs in treatment, as it may be different for BED compared to other forms of ED. This is still an insufficiently investigated topic.

### Shame is reinforced in treatment

The results describe specific ways in which the participants encounter shame reinforcement when seeking help, especially through the misconception of their BED as a weight and lifestyle issue. The focus on weight had been maintained in healthcare settings for many years for all participants. Considering the social emphasis on slimness and self-control, being overweight induces shame in itself [71]. Shame can be seen as related to representations of others negative thoughts of oneself [72–75]. Our findings concur with this understanding, as the participants expect to be judged against societal ideas of body images and eating behaviors, which is also what they experience in healthcare. Being labeled as having a weight problem reinforces their sense of lost control of their eating behavior and body, suggesting that they should be able to regain control through the help they receive in weight loss or lifestyle interventions. This affects how they perceive their own personal abilities and character. This aligns with existing research suggesting that that people with BED experience a global internalized shame [69, 70].

Comments on weight and eating behaviour play a role in the development of BED as seen through the participants subjective point of view. Treatment targeting health and lifestyle change might therefore be experienced as repetitive of a painful relational history. Based on how participants described this as triggering, it is assumable that these experiences increase symptom behavior. This is in line with other qualitative research finding that weight stigma and adverse childhood experiences keep patients in shame driven patterns of binge eating [32].

Previous negative experiences with seeking help may cause an underlying distrust in the therapeutic relationship. For some participants, sharing the problem with other BED patients who can relate, created a safe space. As professional practitioners hold a different status in the practitioner-patient relationship, it may not be experienced as inherently safe. From a mentalization perspective, it can be assumed that patients meet the healthcare system with an internalized expectation of being judged for their BED by others. This indicates that practitioners should actively disconfirm such expectations in order to establish a safe environment in treatment.

### Stigmatizing BED

The participants compared themselves to other EDs, leading to an increased sense of shame of what kind of ED they have. This may be seen in light of how health professionals give AN and BN more attention and general awareness. The results indicate that patients with BED encounter more treatment situations that explicitly induces or enhances shame than for other EDs, where treatment and knowledge is more developed. This may be seen in association with previous research that finds differences in attitudes towards AN, BN, BED, overweight and depression amongst the general population [76], showing that «lack of self-discipline» was associated with BED and overweight more than for any other group. Unconscious weight stigmatizing attitudes towards patients in general somatic treatment are documented amongst healthcare workers [77]. Negative attitudes towards BED patients may also exist amongst healthcare workers given the association with overweight. When participants described experiences of having a «failed» form of ED, it may reflect stigmatization related to a specific condition in psychiatric healthcare, as described in existing literature [76, 78–80].

Studies have found that BED patients often feel misunderstood by health professionals [81], indicating limited knowledge of BED among health professionals. As BED is a relatively recent addition to the diagnostic manuals DSM -5 and ICD-11, it is likely that knowledge amongst practitioners mainly encompass AN and BN. When participants are met with misunderstanding, avoidance or silence about their binge eating and their body image experience, it is experienced as a confirmation that their form of ED is not addressable. This avoidance described in our results may reflect practitioners general lack of knowledge or knowing treatment offers are scarce or non-existent. Furthermore, the participants described feelings of having a «failed» form of ED even in settings of specialized healthcare for ED, where they were to expect something different. This seems to be facilitated when practitioners neglected certain aspects of BED that was actually different from other forms of EDs with a more restrictive symptom presentation. In addition, some aspects common to other EDs, are not addressed, such as body image issues and comparing tendencies. The results show that practitioners in specialized treatment overlook aspects of the disorder that is unique to BED and clinical commonalities with other EDs, both being important to BED patients in treatment.

### The body in treatment

Body shame is experienced as a central aspect of the participants BED, acting as an impediment in their everyday life and potential recovery. The participants express an explicit need to address their body relationship in treatment. In this way, the body image experience may represent a pathway towards building trust in the therapeutic relationship. However, the participants experienced silence from practitioners about this topic, which reinforces their body shame. When body shame is present in the relational treatment setting, it may disturb therapeutic processes relying on trust and safety in the therapeutic relationship. It indicates that showing the patient it is possible to talk about, may be an important correcting emotional experience [82].

Few studies have investigated body image as a specific focus for BED treatment [83]. A qualitative study found positive experiences among BED patients receiving treatment with a specific focus on the relationship with their body [11]. This aligns with our study finding that patients require practitioners help and support to address their body relationship in order to be able to relate to it more themselves. Care must be taken in how this is implemented in treatment: highlighting the subjective bodily experience is critical as opposed to focus on the body as an object to be changed, that has been the main experience in treatment for the participants. Berg, Natvik and Eik-Nes [32] found that BED patients experience feelings of inferiority in relationship and treatment settings, and that a weight-neutral treatment program reduced these feelings of inferiority. This is not considered contradictory to our findings of a general need to therapeutically address body shame and body image in BED treatment. The participants in our study pointed out how body image is addressed frequently in treatment of other EDs, leading to a feeling of inferiority in ED treatment. Working on this through a similar approach may therefore contribute to reduce feelings of inferiority in treatment and offer a correctional experience to previous encounters in healthcare that reinforced body shame.

### Body shame as a maintaining mechanism in BED

The results of this study align with previous research about body image disturbances and body overvaluation as likely involved mechanisms in BED [33–36] and suggest that they may be considered as diagnostic descriptions for BED as for AN and BN. The results also indicate from a qualitative patient perspective that the overevaluation seems related to the ED, concordant with quantitative research findings [37–39]. This study can qualitatively elaborate on research suggesting that higher levels of body overvaluation is associated with poorer treatment outcome [37]. If body shame is a barrier for trust in treatment as our results suggests, this may be a specific trajectory in which the treatment response is impaired. Addressing patients’ relationship with the body may improve treatment outcome.

### Implications for research and practice

Healthcare practitioners must be aware that patients seek help with underlying shameful experiences and previously have considerable negative encounters with the healthcare system that reinforced feelings of shame. Findings suggest that shame, internalized attitudes in the patients and expectations of negative attitudes among others needs to be addressed and disconfirmed actively by practitioners in early encounters. We encourage ensuring empathetic communication skills that validate and counteract patients’ feelings of shame. Implementing training programs for healthcare professionals to recognize and address internalized shame in BED patients may be useful. We further suggest development of protocols for initial patient assessment that include discussions around shame and its impact on eating behaviors.

Information on BED pathology should be included in educational programs for healthcare professions who meet this group of patients in their practice. Awareness of subconscious attitudes and stereotyping towards BED and general weight stigma amongst healthcare practitioners should be further assessed through education.

Group based formats of treatment and support may be a way of helping patients to open up in safe non-judgemental environments. When group formats of treatment or support is considered, separate groups for BED as opposed to mixed groups of EDs is recommended. Practitioners should be aware of the similarities and differences with other forms of EDs and talk about this with patients.

Patients body image and experienced relationship with their body should be addressed and included as a focus of interventions in treatment for BED, as with other EDs. Addressing this is also a specific way to facilitate safety in the therapeutic relationship and reduce feelings of shame that may influence therapeutic alliance and treatment outcome. This study supports the need for further research on body image disturbance as a clinical trait and the consideration for inclusion in the diagnostic description of BED. It implies that further research on how shame and body image work as possible maintaining mechanisms in binge eating behavior is called for.

There is still need for conducting studies on BED in different clinical populations, including different age groups, genders, ethnicities and socio-economic statuses to understand variations in experiences and needs in treatment. Further exploration of gender differences in the manifestation and treatment of BED is encouraged considering the gender distribution of BED and findings on gender differences [53, 84]. Examining the effectiveness of combining different therapeutic approaches for BED (e.g. CBT-ED and mindfulness or compassion focused models) is to our knowledge not executed in the research field yet and may be useful in further assessing treatment approaches for BED.

The participants in this study largely emphasized relational and contextual aspects of care, such as feeling understood and supported over symptom-specific issues like intrusive thoughts around food or loss of control during binge episodes. This may reflect a difference in priorities between treatment focuses and patient—experienced needs, underscoring the importance of integrating patient perspectives into treatment development. As the field of BED treatment continue to grow, incorporating diverse patient experiences can inform the design of interventions that better address both relational and symptom specific challenges.

### Limitations within this study

Homogeneity within the sample in regard to ethnicity, gender and educational level may limit the applicability of these results. The emphasis on body image in this study’s results may have been impacted by gender homogeneity within the sample, as it has been shown that women with BED exhibit more body dissatisfaction and drive for thinness than men with BED [84]. Most participants were recruited through their participation in an inpatient treatment program, which is unusual. We are unsure of how this may have affected the results. However, notable differences between participants recruited from inpatient and outpatient care settings were not observed in our sample.

Homogeneity within the research team in regard to gender and professional background, may also have limited the study and expectations of findings. Given the low sample size, generalization by virtue of a representative sample is not possible. However, a qualitative generalization makes sense, as the findings may be transferable to similar samples or situations [58]. By providing detailed descriptions of the participants and their experiences, it is up to the reader to determine the relevance of the findings in their own settings.

## Conclusion

This investigation sought to elucidate the healthcare needs and experiences of individuals with BED. Significant findings indicate that internalized shame about binge eating and body image is prevalent among the participants, often reinforced by a healthcare system focused on weight and lifestyle. This shame hinders treatment seeking and contributes to the maintenance of the disorder.

Early life experiences and interactions with healthcare providers were identified as sources of lasting shame. This study underscores the necessity for healthcare practitioners to understand and address the complex needs of BED patients to improve treatment outcomes and patient satisfaction. The traditional focus on weight management in treatment is called into question, suggesting a shift towards more patient centered approaches. Group-therapy has the potential to alleviate shame provided that the setting does not include patients with other kinds of EDs, which might exacerbate shame issues. Healthcare providers are encouraged to cultivate a safe space for discussing body image and shame as a core component of effective BED treatment.

In summary, the study advocates for a reorientation of clinical practice to better meet the needs of BED patients, emphasizing empathetic holistic care over weight-centric approaches.

## Supplementary Information

Additional file 1.

## Data Availability

In accordance with privacy concerns and national legislation, qualitative data is not available.

## Abbreviations

BED: Binge eating disorder
ED: Eating disorder
AN: Anorexia nervosa
BN: Bulimia nervosa
TA: Thematic analysis
IPA: Interpretive phenomenological analysis
