# Supplementary material for: “Binge eating disorder is the slum of eating disorders”: a qualitative study of Norwegian women with binge eating disorder in the encounter with the healthcare system
Source: J Eat Disord. 2025 Mar 19;13:51. doi: 10.1186/s40337-025-01223-z (PMC11921570; doi:10.1186/s40337-025-01223-z)
Supplement: Supplementary file 1 — Additional file 1. [file 40337_2025_1223_MOESM1_ESM.docx]

**Interview guide**

**Introduction**

Thank you so much for agreeing to participate in this research project. I aim to investigate how individuals with binge eating disorder have experienced being treated within the healthcare system and how these experiences have influenced their perception of potential for recovery. Since you have personal experience with this, I would like your help in understanding it better from your perspective as a patient. You are free to share whatever you feel comfortable telling me.

When I refer to treatment, I mean all forms of contact with the healthcare system. I am interested in the care you have received at (*name of institution for recruitment to this project*), but also any previous treatment experiences if you wish to share them. Additionally, I will ask you about your experience of living with binge eating disorder itself.

The interview will last approximately 1-1.5 hours. I will first ask you for some brief background information. After that, I will ask you open-ended questions about the study's topic, where I would like you to share your experiences, thoughts, feelings, and reflections. I do not have any prior information about you. If you feel comfortable, you may share stories or examples from your life. It is completely voluntary to answer the questions, and you can withdraw from the interview at any time without giving a reason.

I am going to repeat some of the information contained in the written consent that you have signed, just to make sure that the content is understood. I will record the interview to analyze your responses. The audio file will be handled and stored securely and will only be accessible to those directly involved in the project. The information from the interviews will be fully anonymized in the publication, and you will not be identifiable. Your name will not be made public.

I am bound by confidentiality. The information you share with me will not be shared with anyone other than my project colleagues (Margrete Seeger Halvorsen, KariAnne Vrabel, and Kjersti Solhaug Gulliksen). What you tell me will not affect your access to any treatment options.

**Qualitative Questions**

Now I will move on to the more open-ended questions. Here, I would like you to reflect freely and think aloud; there are no right or wrong answers. I would like to start broadly. If you were to give me an image or a sentence, the first thing that comes to mind, when you think of your experience with the healthcare system, what would you say?

**Experience of mental struggles and eating disorders**

You have received care for binge eating disorder at (*name of institution for recruitment to this project*). How would you describe the issues that led to you receiving this care?
Do you have other mental struggles that you experience as problematic? If so, what are they?

**Treatment experiences**

How would you describe your experience of encountering the healthcare system with your binge eating disorder?
How did you first get in contact with the healthcare system regarding your binge eating disorder?

How have you experienced the treatment or care you have received?

Have you felt that important aspects of your eating issues have been addressed when you’ve been in contact with the healthcare system?

How long had you been dealing with eating problems before you received the diagnosis of binge eating disorder?

How do you feel about being diagnosed with binge eating disorder?

**Possible recovery in relation to treatment experiences**

What do you understand by "recovery" in relation to binge eating disorder?

Do you feel you have recovered in relation to your binge eating disorder?

-If so, in what way?

Would you say that contact with the healthcare system has contributed to your improvement?

-If so, how?

-If not, why not?

Are there other factors, aside from the you’ve received from the healthcare system, that have contributed to your possible recovery?

What could you have needed to recover from your eating disorder?

**Check on Important Topics**

Is there anything important I haven’t asked you about?

Is there anything you think I should ask others with binge eating disorder in order to understand them better?

How has it been for you to participate in the interview?
